# Chronic fetal hypoxia and antenatal Vitamin C exposure differentially regulate molecular signalling in the lung of female lambs in early adulthood

**DOI:** 10.3389/fphys.2024.1488152

**Published:** 2025-01-15

**Authors:** Erin V. McGillick, Sandra Orgeig, Beth J. Allison, Kirsty L. Brain, Melanie R. Bertossa, Stacey L. Holman, Ashley S. Meakin, Michael D. Wiese, Youguo Niu, Nozomi Itani, Katie L. Skeffington, Christian Beck, Kimberly J. Botting-Lawford, Janna L. Morrison, Dino A. Giussani

**Affiliations:** ^1^ Early Origins of Adult Health Research Group, Health and Biomedical Innovation, University of South Australia, Adelaide, Australia; ^2^ Clinical and Health Sciences, University of South Australia, Adelaide, Australia; ^3^ Department of Physiology, Development and Neuroscience, University of Cambridge, Cambridge, United Kingdom

**Keywords:** chronic fetal hypoxia, antenatal antioxidant, Vitamin C, postnatal, molecular, lung

## Abstract

**Introduction:**

Chronic fetal hypoxia is commonly associated with fetal growth restriction and can predispose to respiratory disease at birth and in later life. Antenatal antioxidant treatment has been investigated to overcome the effects of oxidative stress *in utero* to improve respiratory outcomes. We aimed to determine if the effects of chronic fetal hypoxia and antenatal antioxidant administration persist in the lung in early adulthood.

**Methods:**

Chronically catheterised pregnant sheep were exposed to normoxia (N; n = 20) or hypoxia (H; n = 18; 10% O_2_) ± maternal daily i. v. saline (N = 11; H = 8) or Vitamin C (VC; NVC = 9; HVC = 10) from 105 to 138 days (term, ∼145 days). Lungs were collected from female lambs 9 months after birth (early adulthood). Lung tissue expression of genes and proteins regulating oxidative stress, mitochondrial function, hypoxia signalling, glucocorticoid signalling, surfactant maturation, inflammation and airway remodelling were measured.

**Results:**

Chronic fetal hypoxia upregulated lung expression of markers of prooxidant, surfactant lipid transport and airway remodelling pathways in early adulthood. Antenatal Vitamin C normalized prooxidant and airway remodelling markers, increased endogenous antioxidant, vasodilator and inflammatory markers, and altered regulation of hypoxia signalling and glucocorticoid availability.

**Conclusion:**

There are differential effects of antenatal Vitamin C on molecular markers in the lungs of female lambs from normoxic and hypoxic pregnancy in early adulthood.

## 1 Introduction

Chronic fetal hypoxia is commonly experienced in pregnancies complicated by fetal growth restriction (FGR) and can predispose to altered respiratory outcomes at birth and in later life (McGillick et al., 2016a; McGillick et al., 2016b). It is well established that the timing of onset (early versus late gestation), severity (% oxygen reduction) and duration (days, weeks, months) of the chronic hypoxic insult are key regulators underlying either increased, decreased or in some case no change in molecular markers of fetal lung maturation (Morrison, 2008; McGillick et al., 2016b; Rood et al., 2019; Darby et al., 2020; Gonzaléz-Candia et al., 2020a). These findings support the observed heterogeneity in respiratory outcome at birth in growth restricted newborns (McGillick et al., 2016c; Rood et al., 2019; Gonzaléz-Candia et al., 2020b).

Maturation of the endogenous antioxidant system is essential for normal development of the lung and is an important pulmonary defence mechanism in preparation for exposure to the relative hyperoxia at birth (Frank and Sosenko, 1987; Saugstad, 1998; Asikainen and White, 2005). Exposure of the fetus to chronic hypoxia leads to oxidative stress that can have significant effects on the developing organs (Giussani, 2021). During fetal lung development, exposure to chronic hypoxia has significant effects on the molecular markers controlling glucocorticoid availability and receptor signalling and regulation of hypoxia signalling, which alter normal airway development and pulmonary surfactant system maturation (Orgeig et al., 2010; Orgeig et al. 2011; Orgeig et al. 2015; McGillick et al., 2017). One of the most significant regulators is timing of chronic hypoxia onset, with a significant differential effect observed in molecular markers of surfactant maturation between early-onset FGR (placental restriction sheep model) downregulating pathway expression compared with late onset FGR using this model of maternal chronic hypoxia (50% reduction in oxygen) in the last month of gestation, that leads to upregulation of signalling pathways (McGillick et al., 2016a). Alterations to lung molecular signalling pathways regulating the surfactant system by exposure to chronic fetal hypoxia have been proposed as key regulators of newborn respiratory distress risk in FGR pregnancies (McGillick et al., 2016c; McGillick et al., 2016b). Beyond surfactant maturation, studies in sheep have also provided important insights into the effect of chronic hypoxia in fetal life on programming of vascular changes underpinning development of pulmonary hypertension in the fetal and newborn period and adulthood (Liu et al., 2009; Herrera et al., 2010; Hadley et al., 2012; Yang et al., 2012; Rood et al., 2019; Gonzaléz-Candia et al., 2020a; Leslie et al., 2021).

To overcome the effects of chronic hypoxia and oxidative stress on fetal development, maternal treatment with antioxidants in hypoxic pregnancy has been used to protect against cardiovascular dysfunction in the fetal and adult offspring (Herrera et al., 2012; Niu et al., 2013; Herrera et al., 2014; Brain et al., 2015; Brain et al., 2019). In a model of fetal chronic hypoxia at altitude, administration of antioxidant melatonin has positive effects on vascular reactivity and oxidative stress in the lung of newborn lambs (Gonzalez-Candia et al., 2019; Gonzaléz-Candia et al., 2020b). In the normally grown male fetus undergoing healthy normoxic gestation, antenatal Vitamin C treatment for a month in late gestation upregulated the expression of genes important for normal lung development and preparation for exposure to the air-breathing environment at birth (McGillick et al., 2022). These findings included significant effects on molecular markers of antioxidant defence, hypoxia signalling, surfactant maturation and airway remodelling pathways (McGillick et al., 2022). In contrast, exposure to chronic hypoxia during pregnancy also led to upregulation of molecular markers of lung development in male fetuses, but there was limited responsiveness to antenatal Vitamin C exposure in the lung of the hypoxic fetus compared to the normoxic fetus (McGillick et al., 2025). This finding suggested a maximal upregulation of molecular signalling pathways in the fetal lung in response to chronic fetal hypoxia alone (McGillick et al., 2025). In this ovine model of chronic fetal hypoxia and antenatal Vitamin C intervention, it is currently unknown if any effects on lung molecular signalling persist into postnatal life. This is important because understanding the interaction between chronic hypoxia, FGR and antenatal antioxidant exposure on the lung and surfactant maturation in both fetal and postnatal life is essential to target potential therapies to improve respiratory outcomes in complicated pregnancy.

This study investigated molecular markers of pathways involved in oxidative stress, mitochondrial function, antioxidant defences, glucocorticoid signalling, hypoxia signalling, airway remodelling, surfactant maturation and inflammation in the lung of young adult lambs from normoxic or hypoxic pregnancy with and without antenatal Vitamin C treatment. We hypothesized that chronic fetal hypoxia and antenatal Vitamin C exposure would impact factors regulating molecular signalling in the lamb lung in early adulthood.

## 2 Materials and methods

### 2.1 Ethics approval

All animal procedures were approved by the University of Cambridge Ethical Review Board and were performed in accordance with the UK Animals (Scientific Procedures) Act 1986. The animal experiments were conducted at the Barcroft Centre at the University of Cambridge.

### 2.2 Surgery and experimental protocol

At 100 ± 1 day (d) gestation (term, ∼145 days), 38 pregnant Welsh mountain ewes carrying singleton pregnancies underwent a sterile laparotomy under general anesthesia (1.5%–2.0% isofluorane in 60:40 O_2_:N_2_O) to determine fetal sex and catheterisation of the maternal femoral artery and vein, as previously described (Brain et al., 2015; Allison et al., 2016; Brain et al., 2019; Botting et al., 2020). To control for sex differences, only female fetuses were included in this postnatal study. Male fetuses were assigned to fetal studies [Brain et al., 2015; McGillick et al., 2017; McGillick et al., 2022; McGillick et al., 2025].

At 103 d gestation, when the fetal lung is in the canalicular phase of development, ewes were randomly assigned to one of two experimental groups: Normoxic (N; n = 20) or Hypoxic (H; n = 18). Ewes allocated to the Normoxic group remained housed in individual floor pens for the duration of the experimental protocol. Pregnant ewes assigned to the Hypoxic group were housed in bespoke isobaric hypoxic chambers (Telstar Ace, Dewsbury, UK) and exposed to ∼10% O_2_ from 105 d by altering the incoming inspirate mixture as previously described (Brain et al., 2015). While the Control ewes were not housed in the same chambers as the hypoxic group, we controlled for as many factors as possible, including the pen floor surface area, humidity, temperature, noise levels and the feeding regimen (Brain et al., 2015). Critically, animals in both groups could always see other sheep, thereby minimizing stress. Measurements of stress hormones in maternal plasma during chronic hypoxia confirmed a lack of any change from baseline (Brain et al., 2015). This model of maternal chronic hypoxia also does not affect maternal food intake (Brain et al., 2015).

At 105 d gestation, Normoxic and Hypoxic ewes were randomly divided into 2 groups receiving either a daily bolus (between 09:00-10:00) of intravenous Saline (0.6 mL/kg; Normoxic + Saline, N = 11; Hypoxic + Saline, H = 8) or Vitamin C (200 mg/kg i. v. daily Ascorbate; A-5960; Sigma Chemicals, UK; 1.14 mmol/kg/day dissolved in 0.6 mL/kg saline; Normoxic + Vitamin C, NVC = 9; Hypoxic + Vitamin C, HVC = 10) until 135 d gestation. Vitamin C was chosen for administration in this study due to it being a commonly used water-soluble antioxidant supplement that can cross the placenta (Sales et al., 2019) and shows powerful antioxidant protection in the offspring (Thaket al. 2010; Giussani et al., 2012; Richter et al., 2012; Brain et al., 2019). While Vitamin C can be easily administered orally daily in humans, the maternal i. v. route was chosen in this study to ensure complete and controlled delivery into the maternal circulation. The effect of maternal chronic hypoxia and antenatal Vitamin C administration on maternal and fetal blood gas status, cortisol and Vitamin C concentrations have been previously reported (Brain et al., 2015; McGillick et al., 2017; McGillick et al., 2022).

### 2.3 Postnatal care of lambs

At 138 d gestation, pregnant ewes were transferred from the hypoxic chambers to individual pens in a barn (same as Control ewes) with a 12:12-h light–dark cycle, where they were returned to normoxic conditions. Ewes were allowed to deliver naturally, lambs were weighed within 12 h of birth, and they remained with their mothers until weaning at 3 months. Following weaning, lambs were maintained in a farm grazing on grass with free access to water in paddocks that belong to the Barcroft Centre at the University of Cambridge.

### 2.4 Post-mortem and sample collection

At 9 months after birth, past sexual maturity and into early adulthood, lambs were euthanised by an overdose of sodium pentobarbitone (0.4 mL/kg, intravenous administration, Pentoject; Animal Ltd., York, UK). Body and organ weights were recorded. A piece of left lung tissue was snap-frozen in liquid nitrogen and stored at −80°C for measuring hormone concentrations, and gene and protein expression analysis. A section of right lung tissue was immersion fixed in 4% paraformaldehyde and processed to paraffin for immunohistochemical analysis.

The tissues generated in this study were part of a programme of work designed with the primary objective of investigating cardiovascular physiology (Brain et al., 2019). This study used the tissues generated in a subset of the cohort to address additional scientific questions retrospectively. This scientific approach is strongly promoted by the UK Home Office 3R principle of Replacement, Reduction and Refinement, thereby making best use of the valuable experimental material (Russell and Burch, 1959). Consequently, no prospective pulmonary functional outcomes were performed, and the lung tissue collected was immersion fixed rather than perfusion fixed.

### 2.5 Quantification of hormone concentration in lung tissue

Lamb lung hormone concentrations (N = 11, NVC = 9, H = 8, NVC = 10) were determined by liquid chromatography (LC; Shimadzu Nexera XR, Shimadzu, Japan) coupled to a SCIEX 6500 Triple-Quad tandem mass spectrometry system (MS/MS; SCIEX, US) using an adapted protocol (McBride et al., 2020; Dimasi et al., 2023). Lung tissue hormones measured were cortisol, cortisone, triiodothyronine (T3) and thyroxine (T4). Initially, lung tissue was homogenised in 500 μL 0.9% NaCl at 50 h/z for 2 min and then centrifuged at 16,000 *g* for 10 min at 4°C. 100 μL of supernatant was added to 300 μL acetonitrile containing 50 ng/mL internal standard (cortisol-9,11,12,12-d4; Toronto Research Chemicals, Toronto, Canada), vortexed for 1 min and then centrifuged at 12,000 *g* for 10 min. Supernatant was transferred to a fresh Eppendorf tube and the remaining pellet was resuspended in 300 μL ethyl acetate, vortexed for 1 min and then centrifuged at 12,000 *g* for 10 min. Supernatant was added to the acetonitrile, mixed by inversion, and then evaporated to dryness using the GeneVac EZ-2 Evaporating System (GeneVac, UK). Dried samples were reconstituted in 50% methanol and then injected onto an ACQUITY UPLC BEH C18 Column 130Å, 1.7 µm, 2.1 mm × 100 mm (Waters Corp, US). Mobile phases were 0.1% formic acid in water (A) and 0.1% formic acid in acetonitrile (B). Flow rate was 0.3 mL/min and mobile phase B was initially 10% and increased linearly to 90% over 10 min and then held at 90% for 2 min, after which it returned to 10% over 30°and then held for 3 min prior to injection of the next sample. Hormone concentrations were calculated via integration with a standard curve. Data are presented indexed to lung tissue weight. Data is presented as individual hormone concentrations as well as ratios of cortisol/cortisone and T4/T3.

### 2.6 Quantification of lung mRNA expression

RNA was extracted and cDNA synthesized from lamb lung tissue samples (∼50 mg; NS = 11; NVC = 9; HS = 8; HVC = 10) using QIAzol Lysis Reagent Solution and Qiagen miRNeasy purification columns (Qiagen, Victoria, Australia) and cDNA was synthesised using Superscript III First Strand Synthesis System (Invitrogen, Carlsbad, CA, United States) as previously described (Soo et al., 2012; McGillick et al., 2013; Lie et al., 2014; McGillick et al., 2022). The expression of target genes regulating oxidative stress, antioxidant defences, hypoxia signalling, glucocorticoid signalling, surfactant maturation (protein and lipid synthesis), inflammation and airway remodelling were measured by quantitative reverse transcription polymerase chain reaction (qRT-PCR; Table 1), as previously described (Soo et al., 2012; McGillick et al., 2013; Lie et al., 2014; McGillick et al., 2022). Gene expression was measured by qRT-PCR using Fast SYBRR Green Master Mix (Applied Biosystems, Foster City, CA, United States) in a final volume of 6 μL on a ViiA7 Fast Real-time PCR system (Applied Biosystems) as described previously (McGillick et al., 2013; McGillick et al., 2015; McGillick et al., 2016b). The abundance of each transcript relative to the abundance of stable reference genes (beta-actin, peptidylprolyl isomerase, tyrosine 3-monooxygenase) was calculated using DataAssist 3.0 analysis software and is expressed as mRNA mean normalized expression (MNE) ± SEM (Soo et al., 2012; McGillick et al., 2013; Lie et al., 2014; McGillick et al., 2022).

**TABLE 1:** Target genes regulating oxidative stress, hypoxia signalling, glucocorticoid signalling, surfactant maturation, inflammation and airway remodelling by quantitative real-time RT-PCR [all primer sequences and concentrations previously published (McGillick et al., 2017)].

	Gene name	Protein name	Function
Oxidative stress			
Nicotinamide adenine dinucleotide phosphate oxidase	*NOX-4*	NAPDH oxidase 4	Pro-oxidant marker
Heme oxygenase-1	*HMOX-1*	HMOX-1	Pro-oxidant marker and vasodilator
Superoxide dismutase-1	*SOD-1*	SOD-1	Antioxidant marker
Catalase	*CAT*	CAT	Antioxidant marker
Hypoxia signalling			
Hypoxia inducible factor alpha subunits	*HIF-1α* *HIF-2α*	HIF-1α HIF-2α	Major regulator of hypoxia signaling
Egl-9 family hypoxia-inducible factor enzymes (encoding the prolyl hydroxylase domain proteins)	*EGLN-1* *EGLN-2* *EGLN-3*	PHD-2 PHD-1 PHD-3	Regulator of HIF activity and signaling
Vascular endothelial growth factor	*VEGF*	VEGF	Hypoxia responsive gene and vasodilator
Adrenomedullin	A*DM*	ADM	Hypoxia responsive gene and vasodilator
Lysine (K)-specific demethylase 3A	*KDM3A*	JMJD1A	Hypoxia responsive gene
Glucocorticoid signalling			
11β-hydroxysteroid dehydrogenase-1	*HSD11B-1*	11βHSD-1	Glucocorticoid activating enzyme isoform
11β-hydroxysteroid dehydrogenase-2	*HSD11B-2*	11βHSD-2	Glucocorticoid de-activating enzyme isoform
Glucocorticoid receptor	*NR3C1*	GR	Cellular glucocorticoid receptor
Inflammation			
Transforming growth factor beta	*TGFB1*	TGF-β	Inflammatory marker
Interleukin-1 beta	*IL-1B*	*IL1-*β	Inflammatory marker
Surfactant maturation and lipid transport			
Surfactant protein	S*FTP-A* S*FTP-B* S*FTP-C* S*FTP-D*	SP-A SP-B SP-C SP-D	Pulmonary immunity and surface tension regulating
Phosphate cytidylyltransferase 1, choline, alpha	*PCYT1A*	PCYT1A	Surfactant lipid synthesis
ATP-binding cassette, sub-family A (ABC1), member 3	*ABCA3*	ATPA3	Surfactant lipid transport
Airway remodelling			
Elastin	*ELN*	ELN	Structural role in lung tissue development
Collagen type 1 alpha 1	*COL1A1*	COL	Structural role in lung tissue development
Matrix metallopeptidase 9	*MMP-9*	MMP-9	Structural role in alveolar development

### 2.7 Quantification of lung protein expression

Protein was extracted by sonication of lamb lung tissue (∼100 mg, NS n = 6, NVC n = 7, HS n = 7, NVC n = 7) and protein content determined by a MicroBCA Protein Assay Kit (PIERCE, Thermo Fisher Scientific Inc., Rockford, Illinois) as previously described (Darby et al., 2019; McGillick et al., 2022). Extracted protein samples (75 μg) were subject to sodium dodecyl surface (SDS) page and stained with Coomassie blue to determine equal protein loading. Protein samples were transferred onto a 0.45 µm nitrocellulose membrane (Hybond ECL, GE Healthcare, NSW, Australia), and then stained with Ponceau S (0.5% Ponceau in 1% acetic acid) in order to determine the efficacy of the transfer. The membranes were briefly washed with 7% acetic acid followed by a reverse osmosis water rinse, and then imaged for Ponceau S using ImageQuant LAS4000 (GE Healthcare, Victoria, Australia). Following imaging, membranes were washed 3 × 5 min in tris-buffered saline (TBS). The membranes were blocked in 5% bovine serum albumin (BSA) in TBS with 1% Tween-20 (TBS-T) for 1 h at room temperature. The membranes underwent 3 × 5 min washes in TBS-T and were incubated with the primary antibody overnight at 4°C.

Primary antibodies of interest were Mitobiogenesis Western Blot Cocktail (MITOBIO: 1:250, in 5% BSA in TBST, #ab123545, Abcam; 70, 42 (β-actin internal loading control), 35 kDa), OxPhos Rodent Western blot Antibody Cocktail (1:500, in 5% BSA in TBST, #ab110413, Abcam, 20, 30, 40, 48, 55 kDa band), Catalase (1:1,000, in 5% BSA in TBS-T, #14097S, Cell Signalling Technology; 60 kDa band), PHD-1 (1:1,000, in 5% BSA in TBS-T, #ab108980, Abcam; 44 kDa band), 11βHSD-1 (1:1,000, in 5% BSA in TBS-T, #ab39364, abcam; 40 kDa band), ABCA3 (1:500, in 5% BSA in TBS-T, #WRAB-ABCA3, Seven Hills Bioreagents; 130 kDa band) and Elastin (1:1,000, in 5% BSA in TBS-T, #ab213720, Abcam; 68 kDa band). All antibodies were validated by testing multiple dilutions to identify proteins of interest at their respective kDa bands without non-specific staining by our group in sheep tissue. Protein expression determined using the MITOBIO, OxPhos, PHD-1 and 11βHSD-1 antibodies have been published previously by our group (Orgeig et al., 2015; Darby et al., 2019; Dimasi et al., 2023). The commercially available ABCA3 and Catalase antibodies were acquired specifically for this study and the kDa bands analyzed were cross-checked with the expected kDa from the antibody supplier and previous studies using the same antibody in a similar context. Apart from mitochondrial function antibodies, the remaining primary antibodies were chosen based on genes that changed in response to chronic fetal hypoxia and/or antenatal Vitamin C (McGillick et al., 2017; McGillick et al., 2022) to determine if the transcriptional changes observed translated into protein abundance differences. Following incubation with the primary antibody, the blots were washed and incubated with the relevant species of Horse Radish Peroxidase labelled secondary IgG antibody for 1 h at room temperature. Enhanced chemiluminescence using SuperSignal West Pico Chemiluminescent Substrate (Thermo Scientific, Australia) was used to detect the blots. The Western blot was imaged using ImageQuant LAS4000 and the protein abundance was quantified by densitometry using Image quant software (GE Healthcare, Victoria, Australia). Total target protein abundance was then normalized to Ponceau S (total protein stain) or reference protein Vinculin (1:2000, in 5% BSA IN TBST, XP HRP conjugate, #18799S, Cell Signalling Technology; 140 kDa band). Mitochondrial abundance was determined via a ratio of mitochondria DNA-encoded COX-I to nuclear DNA-encoded COXII as previously described (Dimasi et al., 2023).

### 2.8 Immunohistochemistry and quantification of surfactant producing cells within lung tissue

To determine the effect of chronic fetal hypoxia and antenatal Vitamin C administration on the surfactant producing capacity of the postnatal lung at the structural level, immunohistochemistry was performed (NS = 5; NVC = 6; HS = 5; HVC = 6) using a rabbit anti-human mature surfactant protein B (SP-B) antibody (1:500, WRAB-48604, Seven Hills Bioreagents, Ohio), as previously described (McGillick et al., 2017; McGillick et al., 2022). In short, lung tissue slides (5 µm thickness) were baked at 60°C for 1 h followed by deparaffinization and rehydration. After rehydration, endogenous peroxide solution activity was blocked with 3% hydrogen peroxide (Sigma-Aldrich; St. Louis, MO), followed by heat-induced antigen retrieval in citrate buffer (pH 6.0). Slides were incubated overnight with a primary antibody at 4°C following incubation with non-immune serum (serum blocking solution; Histostain-Plus Kit; Invitrogen, Carlsbad, CA) to prevent non-specific binding. Negative control slides with the primary antibody omitted were used to demonstrate no nonspecific binding of the secondary antibody or reagent contamination (Figure 4A). In addition, negative control slides where incubation with primary antibody was substituted for rabbit serum (Sigma-Aldrich) at the same protein concentration as the diluted primary antibody (Figure 4B) (1:500) was carried out to highlight primary antibody specificity in the positive control slides in the alveolar epithelium observed in this study. A Histostain-Plus kit (Invitrogen) was used with horseradish peroxidase and Histostain-Plus broad spectrum 3,3′-diaminobenzidine chromagen for visualization of the SFTP-B positive cells. All sections were counterstained with Mayer’s hematoxylin (Sigma-Aldrich).

Stained lung tissue sections were examined using Visiopharm new Computer Assisted Stereological Toolbox (NewCAST) software (Visiopharm, Hoersholm, Denmark). Positively staining cells were identified by their cuboidal shape and location in the alveolar epithelium (Figures 4C–F). Analysis was carried out by a trained individual who was blinded to treatment groups. Sixty counting frames (×600 magnification) of the alveolar epithelium were randomly selected per section. Point counting with an unbiased counting frame with an area of 20,000 μm^2^ was used to determine the numerical density of SFTP-B positive cells present in the alveolar epithelium of lung tissue, as previously described (Lock et al., 2015; McGillick et al., 2017; McGillick et al., 2022).

### 2.9 Statistical analyses

All statistical analyses were performed using GraphPad Prism v8. All data were evaluated for outliers ± 2 SD from the mean for each treatment group. All data were tested for normality and transformed if required. Data were analysed using a Two-Way ANOVA for main effects of treatment (Normoxic vs. Hypoxic) and drug (Saline vs. Vitamin C) and an interaction between factors (treatment x drug). When a significant interaction was detected between the two factors (*CAT* and *HIF-2a* gene expression), data were split by treatment to determine the effect of drug administration (S vs. VC) in Normoxic or Hypoxic lambs using the Students’ unpaired *t*-test. All data are presented as mean ± SEM. For all comparisons, *P* < 0.05 was considered statistically significant.

## 3 Results

### 3.1 Fetal and postnatal growth

There was a significant effect of treatment with lambs exposed to chronic fetal hypoxia for a month in late gestation having reduced body weight at birth compared to lambs from normoxic pregnancies (Table 2). Conversely, there was no significant effect of exposure to chronic fetal hypoxia or antenatal Vitamin C on body and organ measures at 9 months of age (Table 2).

**TABLE 2:** Effect of chronic fetal hypoxia and antenatal Vitamin C for a month in late gestation on body and organ weights and lung tissue hormones in early adulthood.

	Normoxic + saline	Normoxic + Vitamin C	Hypoxic + saline	Hypoxic + Vitamin C
Number of lambs	11	9	8	10
Birth weight (Kg)	3.50 ± 0.11	3.43 ± 0.21	3.14 ± 0.11^#^	2.89 ± 0.16^#^
Body weight at 9 months (Kg)	24.50 ± 1.82	28.67 ± 1.13	29.53 ± 1.66	27.35 ± 2.04
Crown-rump length (cm)	85.9 ± 2.5	89.48 ± 1.5	91.6 ± 2.0	89.1 ± 0.9
BMI (Kg/cm^2^)	34.1 ± 1.3	34.6 ± 1.0	35.5 ± 2.4	36.1 ± 2.3
Ponderal index (Kg/cm^3^)	38.6 ± 2.4	37.3 ± 1.0	39.2 ± 3.3	40.6 ± 2.7
Relative brain weight (g/Kg)	3.21 ± 0.15	2.94 ± 0.12	3.11 ± 0.13	3.22 ± 0.19
Relative lung weight (g/Kg)	9.57 ± 0.46	9.89 ± 0.16	11.60 ± 1.33	10.08 ± 0.95
Relative liver weight (g/Kg)	14.56 ± 0.57	16.17 ± 0.82	14.23 ± 1.46	14.68 ± 0.94
Lung tissue cortisol (pg/mg)	0.19 ± 0.01	0.22 ± 0.01	0.21 ± 0.02	0.25 ± 0.02
Lung tissue cortisone (pg/mg)	0.09 ± 0.03	0.02 ± 0.004	0.04 ± 0.01	0.06 ± 0.02
Lung tissue cortisol/cortisone	6.89 ± 2.14	13.62 ± 4.0	4.61 ± 0.91	5.26 ± 1.41
Lung tissue triiodothyronine (T3; pg/mg)	0.0084 ± 0.0004	0.0087 ± 0.0004	0.0078 ± 0.0005	0.0093 ± 0.0003
Lung tissue thyroxine (T4; pg/mg)	0.12 ± 0.02	0.08 ± 0.01	0.10 ± 0.02	0.14 ± 0.02
Lung tissue T4/T3	13.69 ± 2.07	9.81 ± 1.46	11.17 ± 2.30	15.43 ± 2.92

Data expressed as mean ± SEM. Data were analyzed by Two-Way ANOVA, for main effects (treatment or drug) and interaction (treatment x drug). *P* < 0.05 was considered significant. # = significant main effect of treatment (Normoxic vs. Hypoxic). Lung tissue hormone concentrations are presented as pg indexed to mg of tissue used for extractions.

### 3.2 Expression of markers regulating oxidative stress, hypoxia signalling and vasodilation

**FIGURE 1:**
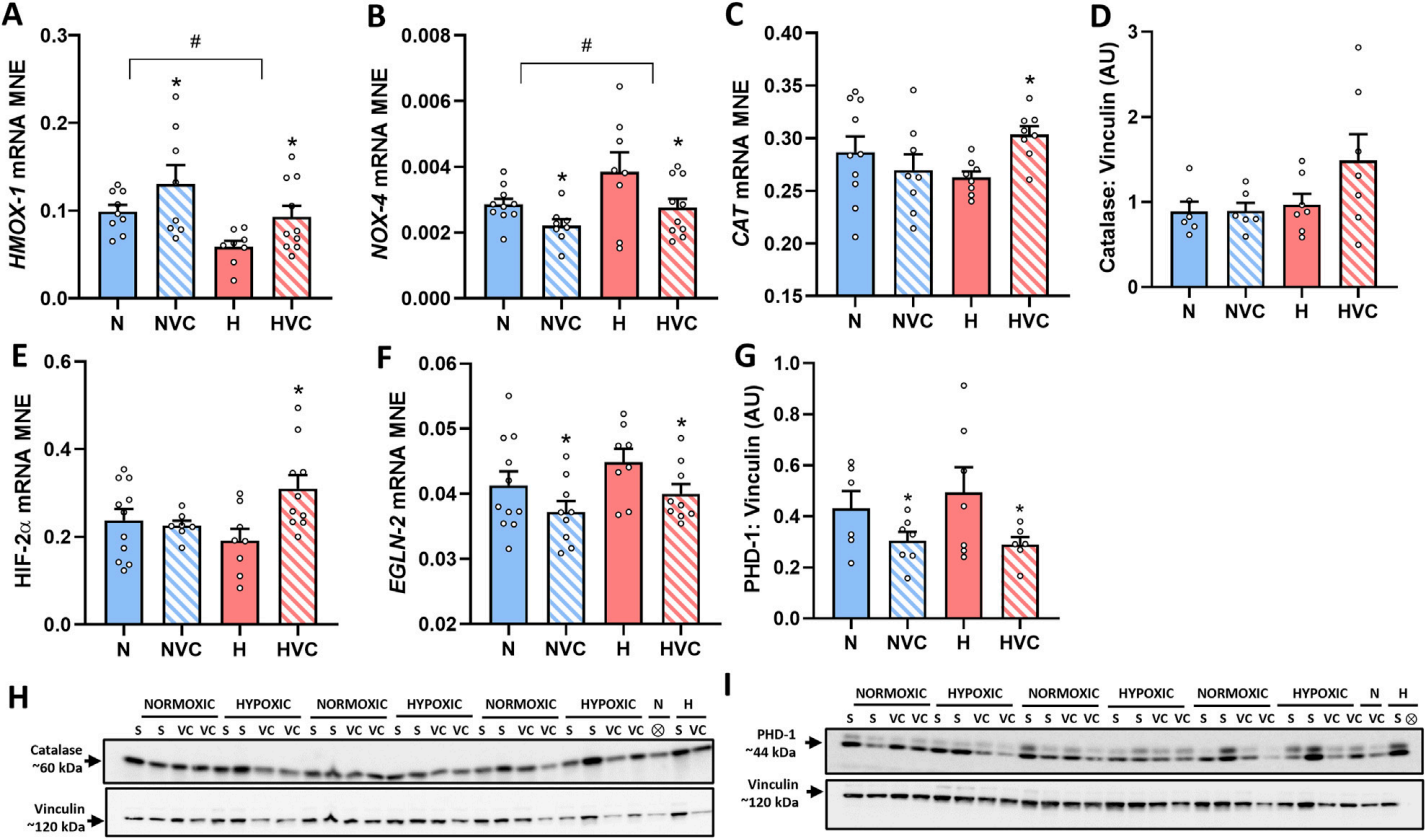
Effect of chronic fetal hypoxia and antenatal Vitamin C on expression of vasodilator pathway and oxidative stress markers [*HMOX-1* **(A)** and *NOX-4* **(B)**], antioxidant defence [*CAT* gene **(C)** and Catalase protein [**(D)**; 60 kDA band on blot in **(H)**]], regulators of hypoxia signalling and feedback [*HIF-2α* **(E)**, *EGLN-2* gene **(F)** and PHD-1 protein **(G)**; 44 kDa band; band 2 on blot in **(I)**] in the lung of young adult lambs. Data expressed as mRNA mean normalized expression (MNE) or mean protein expression in arbitrary units (AU) ± SEM. Normoxic (N) in blue bars and Hypoxic (H) in red bars. Saline exposed lambs in open bars (N and H) and Vitamin C exposed lambs (NVC and HVC) in hashed bars. Western blot images **(H, I)** represent target protein and reference protein for Saline (S) and Vitamin C (VC) lambs in Normoxic (N) and Hypoxic (H) groups. ⊗ = Outlier ± 2 SD from the treatment group mean not included in analysis for this study or excluded due to technical error in the Western blot procedure (last well). Reference protein Vinculin (**H, I**; 120 kDa band) is obtained from the same gel. Data were analyzed by Two-Way ANOVA for main effects (treatment or drug) and interaction (treatment x drug). When a significant interaction was detected, data was split to determine the effect of drug administration in Normoxic and Hypoxic lambs independently using the Students’ unpaired *t*-test. *P* < 0.05 was considered statistically significant. # = effect of treatment (Normoxic vs. Hypoxic) and * = effect of drug (Saline vs. Vitamin C). All significant results presented are main effects, with the exception of *CAT* and *HIF-2α* both of which had a significant interaction and further analysis determined significant effect of Vitamin C in the lung of H lambs only.

Chronic fetal hypoxia significantly reduced *HMOX-1* expression (Figure 1A), while it increased *NOX-4* mRNA expression in the lung of young adult lambs (Figure 1B). Antenatal Vitamin C treatment in hypoxic pregnancy normalized these changes (Figures 1A, B). Antenatal Vitamin C treatment in normoxic pregnancy also increased *HMOX-1* (Figure 1A) and reduced *NOX-4* mRNA expression (Figure 1B). There was no effect of chronic fetal hypoxia or antenatal Vitamin C on *SOD-1* mRNA expression in the lungs of young adult lambs (Table 3). There was a significant effect of antenatal Vitamin C in hypoxic pregnancy, increasing *CAT* mRNA expression in the lung of young adult lambs (Figure 1C), but no significant effect on *CAT* protein expression (Figure 1D).

**TABLE 3:** Effect of chronic fetal hypoxia and antenatal Vitamin C exposure for a month in late gestation on gene expression of markers regulating oxidative stress, hypoxia signalling and feedback, glucocorticoid signalling, inflammation, surfactant protein and lipid synthesis and airway remodelling in the lung of young adult lambs.

	Normoxic + saline (n = 11)	Normoxic + Vitamin C (n = 9)	Hypoxic + Saline (n = 8)	Hypoxic + Vitamin C (n = 10)
Oxidative stress				
*SOD-1*	0.44 ± 0.02	0.45 ± 0.01	0.45 ± 0.04	0.48 ± 0.03
Hypoxia signaling/feedback and vasodilatation				
*HIF-1α*	0.052 ± 0.002	0.051 ± 0.002	0.060 ± 0.005	0.057 ± 0.004
*EGLN1*	0.056 ± 0.002	0.060 ± 0.002	0.058 ± 0.003	0.059 ± 0.002
*EGLN3*	0.0047 ± 0.002	0.0036 ± 0.0003	0.0044 ± 0.0003	0.0043 ± 0.0002
*VEGF*	0.18 ± 0.02	0.19 ± 0.01	0.18 ± 0.02	0.24 ± 0.02
A*DM*	0.021 ± 0.001	0.023 ± 0.003	0.024 ± 0.004	0.021 ± 0.001
*KDM3A*	0.036 ± 0.001	0.038 ± 0.002	0.037 ± 0.002	0.040 ± 0.001
Glucocorticoid signalling				
*HSD11B-2*	0.0011 ± 0.0001	0.0010 ± 0.0001	0.0013 ± 0.0001	0.0012 ± 0.0001
*NR3C1*	0.087 ± 0.005	0.091 ± 0.005	0.088 ± 0.005	0.093 ± 0.005
Inflammation				
*TGFB1*	0.040 ± 0.003	0.042 ± 0.003	0.041 ± 0.002	0.041 ± 0.003
Surfactant protein and lipid synthesis				
S*FTP-A*	2.03 ± 0.07	2.04 ± 0.19	2.08 ± 2.4	2.20 ± 0.24
S*FTP-B*	1.47 ± 0.09	1.34 ± 0.08	1.56 ± 0.12	1.42 ± 0.11
S*FTP-C*	5.76 ± 0.31	5.41 ± 0.26	6.16 ± 0.65	5.16 ± 0.29
S*FTP-D*	0.08 ± 0.01	0.08 ± 0.01	0.07 ± 0.01	0.05 ± 0.01
*PCYT1A*	0.029 ± 0.001	0.0031 ± 0.001	0.029 ± 0.001	0.030 ± 0.001
Airway remodelling				
*COL1A1*	0.28 ± 0.04	0.25 ± 0.02	0.36 ± 0.06	0.29 ± 0.03
*MMP-9*	0.0039 ± 0.0010	0.0026 ± 0.0004	0.0041 ± 0.0007	0.0036 ± 0.0003

Data expressed as mean ± SEM. Data were analyzed by Two-Way ANOVA, for main effects (treatment or drug) and interaction (treatment x drug). *P* < 0.05 was considered statistically significant.

There was no effect of chronic fetal hypoxia or antenatal Vitamin C on expression of *HIF-1α*, *EGLN-1* or *EGLN-3* gene expression in the lung of young adult lambs (Table 3). Antenatal Vitamin C exposure upregulated *HIF-2α* gene expression in the lung of lambs exposed to chronic fetal hypoxia (Figure 1E). Antenatal Vitamin C exposure downregulated *EGLN-2* gene (Figure 1F) and PHD-1 protein expression in the lung of young adult lambs of normoxic and hypoxic pregnancy (Figure 1G). There was no significant effect of chronic fetal hypoxia or antenatal Vitamin C on downstream signalling through genes with hypoxia-responsive elements including *VEGF*, *ADM* and *KDM3A* (Table 3).

### 3.3 Protein expression of markers regulating mitochondrial function

**FIGURE 2:**
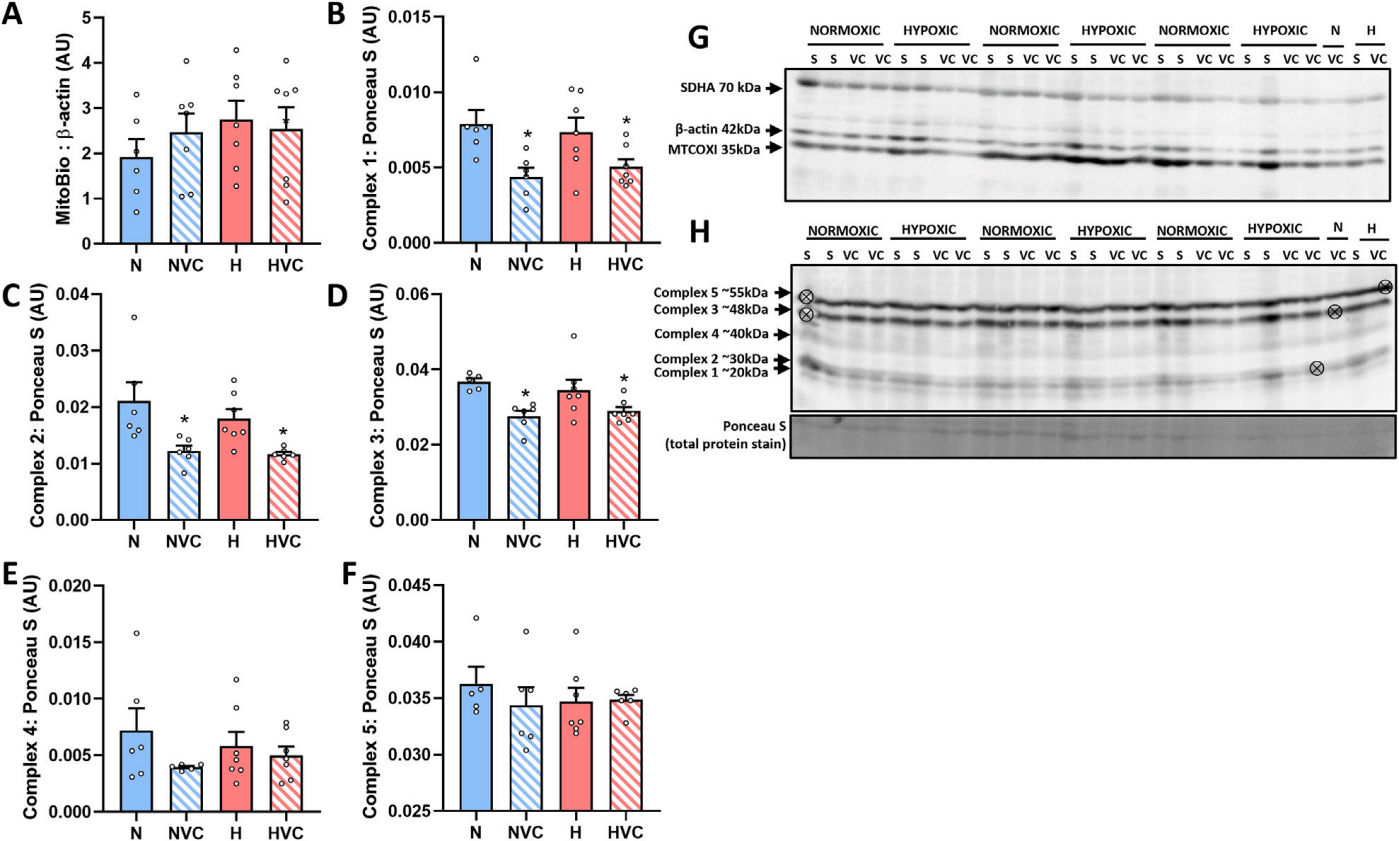
Protein expression of mitochondrial function markers in the lung of young adult lambs following exposure to chronic fetal hypoxia and antenatal Vitamin C exposure. Data presented as normalized protein expression in arbitrary units (AU) for mitochondrial abundance [MITOBIO **(A)**; 35 kDa band on blot **(G)**] and OXPHOS [[complex 1 **(B)**; 20 kDa band on blot **(H)**], [complex 2 **(C)**; 30 kDa band on blot **(H)**], [complex 3 **(D)**; 48 kDa band on blot **(H)**], [complex 4 **(E)**; 40 kDa band on blot **(H)**], [complex 5 **(F)**; 55 kDa band on blot **(H)**]]. Western blot images represent target protein and reference protein for Saline (S) and Vitamin C (VC) lambs in Normoxic (N) and Hypoxic groups (H). ⊗ = Outlier ± 2 SD from the treatment group mean not included in analysis for this study. Reference protein Beta actin [ß-actin Mitobiogenesis Western Blot Cocktail internal loading control; **(G)**; 42 kDa band], and Ponceau S [total protein stain, **(H)**] are obtained from the same gel. Data were analyzed by Two-Way ANOVA for main effects (treatment or drug) and interaction (treatment x drug). *P* < 0.05 was considered statistically significant. # = effect of treatment (Normoxic vs. Hypoxic) and * = effect of drug (Saline vs. Vitamin C). All significant results presented are main effects.

There was no effect of chronic fetal hypoxia or antenatal Vitamin C exposure on mitochondrial abundance protein expression (Figure 2A). There was a significant effect of antenatal Vitamin C exposure, reducing expression of oxidative phosphorylation (OXPHOS) complex 1, 2 and 3 (Figure 2B–D), but no effect on complexes 4 or 5 (Figures 2E, F) in the lung of young adult lambs.

### 3.4 Lung tissue hormone concentration

Lung tissue corticosterone, 11-deoxycortisol and progesterone concentrations were below the limit of quantitation (LOQ; corticosterone LOQ = 0.1 ng/mL, 11deoxycortisol LOQ = 0.5 ng/mL, progesterone LOQ = 0.05 ng/mL). There was no significant effect of chronic fetal hypoxia or Vitamin C exposure on lung tissue cortisol, cortisone, ratio of cortisol/cortisone, thyroid hormone triiodothyronine (T3), thyroid hormone Thyroxine (T4) or ratio of T3/T4 concentrations (Table 2).

### 3.5 Expression of factors regulating glucocorticoid availability and activity

**FIGURE 3:**
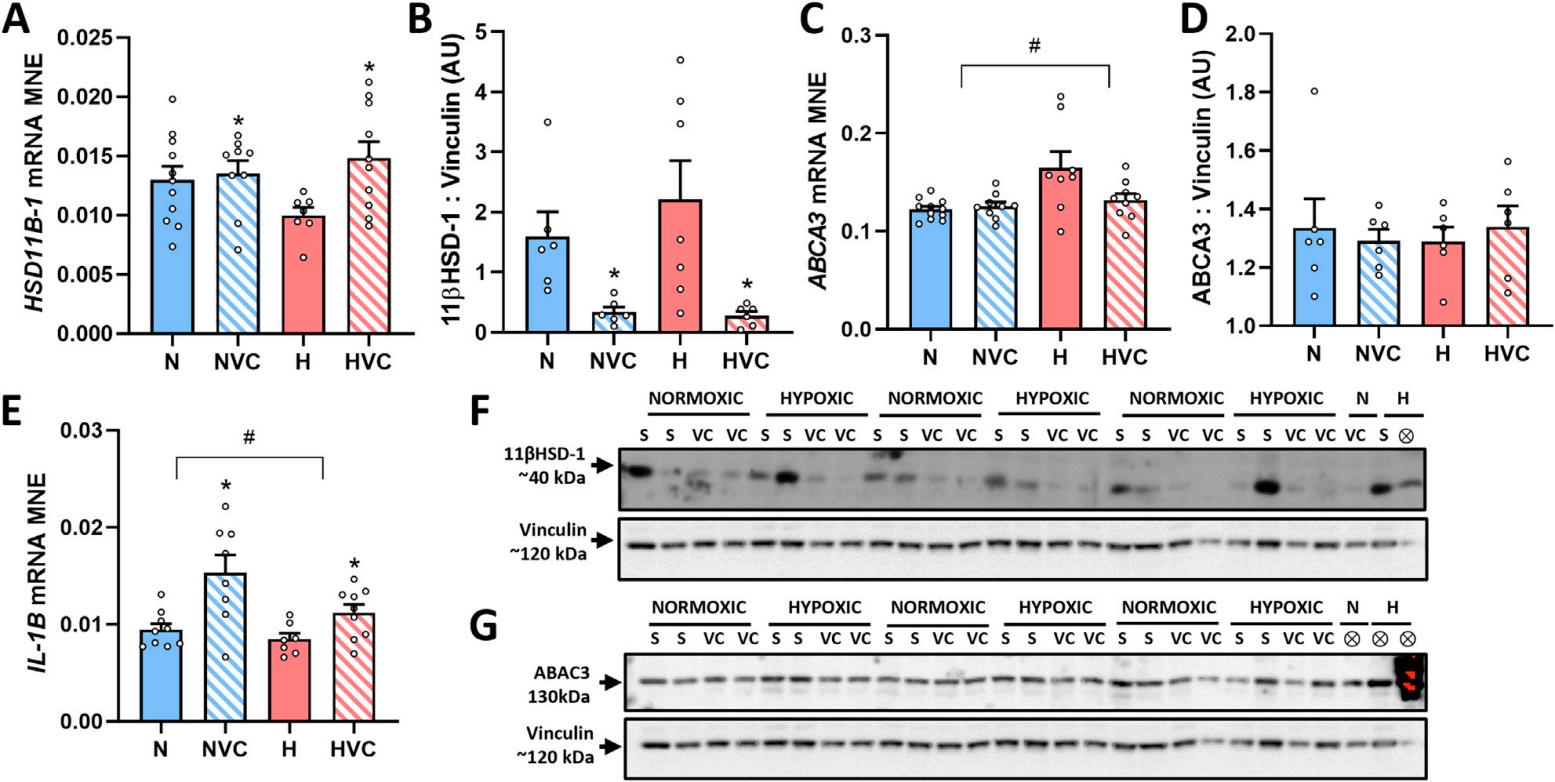
Effect of chronic fetal hypoxia and antenatal Vitamin C on expression of factors regulating glucocorticoid availability [*HSD11B1* gene, **(A)**; 11βHSD-1 protein, **(B)**, 40 kDa band on blot **(F)**], surfactant lipid transport [*ABCA3* gene **(C)**; ABCA3 protein **(D)**, 130 kDa band on blot **(G)**] and inflammation [*IL-1B* gene, **(E)**] in the lung of young adult lambs. Data expressed as mRNA mean normalized expression (MNE) or mean protein expression in arbitrary units (AU) ± SEM. Normoxic (N) in blue bars and Hypoxic (H) in red bars. Saline exposed lambs in open bars (N and H) and Vitamin C exposed lambs (NVC and HVC) in hashed bars. Western blot images represent target protein and reference protein for Saline (S) and Vitamin C (VC) lambs in Normoxic (N) and Hypoxic (H) groups. ⊗ = Outlier ± 2 SD from the treatment group mean not included in analysis for this study or excluded due to technical error in the Western blot procedure (last well). Reference protein Vinculin (120 kDa band) is obtained from the same gel that was used to determine expression of both 11βHSD-1 **(F)** and ABCA3 **(G)**. Data were analyzed by Two-Way ANOVA for main effects (treatment or drug) and interaction (treatment x drug). When a significant interaction was detected, data was split to determine the effect of drug administration in Normoxic and Hypoxic lambs independently using the Students’ unpaired *t*-test. *P* < 0.05 was considered statistically significant. # = effect of treatment (Normoxic vs. Hypoxic). * = effect of drug (Saline vs. Vitamin C). All significant results presented are main effects.

Exposure to antenatal Vitamin C increased glucocorticoid activating enzyme *HSD11B-1* gene expression (Figure 3A) and decreased 11β HSD-1 protein expression in the lung of young adult lambs (Figure 3B). There was no effect of chronic fetal hypoxia or antenatal Vitamin C on mRNA expression of glucocorticoid de-activating enzyme *HSD11B-2* or the glucocorticoid receptor *NR3C1* (Table 3).

### 3.6 Expression of genes regulating surfactant maturation

There was no significant effect of chronic fetal hypoxia or antenatal Vitamin C on mRNA expression of the surfactant proteins or lipid transporter *PCYT1A* in the lung of young adult lambs (Table 3). Exposure to chronic fetal hypoxia significantly upregulated surfactant lipid transporter *ABCA3* mRNA expression (Figure 3C) but had no significant effect on protein expression in the lung of young adult lambs (Figure 3D).

### 3.7 Expression of genes regulating inflammation in the lung

There was a significant decrease in *IL-1B* mRNA expression in the lung of young adult lambs exposed to chronic fetal hypoxia, while exposure to antenatal Vitamin C increased its expression compared to saline lambs (Figure 3E). There was no effect of exposure to chronic fetal hypoxia or antenatal Vitamin C on the expression of *TGFB1* (Table 3).

### 3.8 Markers of surfactant producing capability and airway remodelling

**FIGURE 4:**
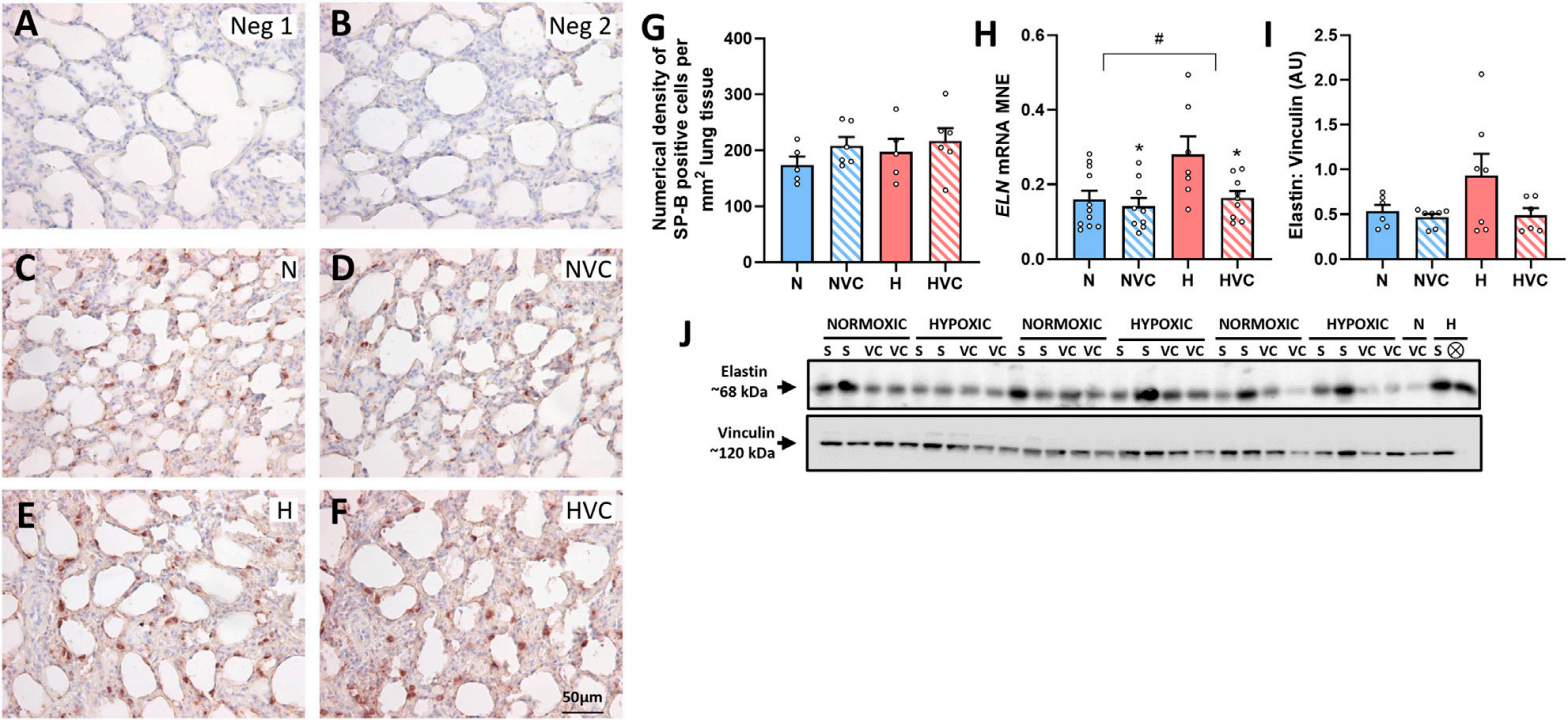
Effect of chronic fetal hypoxia and antenatal Vitamin C on the structure of the lung in young adult lamb. Micrographs (Scale bar = 50 μm) demonstrating no primary antibody negative control **(A)**, 1:500 rabbit serum negative control **(B)**, SP-B immunoreactivity (brown intracellular precipitate) in the alveolar epithelium in the lung of Normoxic + Saline [**(C)**; N, blue bars], Normoxic + Vitamin C [**(D)**; NVC, blue hashed bars], Hypoxic + Saline [**(E)**; H, red bars], Hypoxic + Vitamin C [**(F)**; HVC, red hashed bars] lambs. Numerical density of SP-B cells is expressed as mean ± SEM **(G)**. Expression of *ELN* gene [**(H)**; presented as mRNA mean normalized expression (MNE) ± SEM] and elastin protein [**(I)**; presented as mean protein expression in arbitrary units (AU) ± SEM; 68 kDa band] in the postnatal lung. Western blot image represents target protein **(J)** and reference protein (Vinculin, 120 kDa band) obtained from the same gel. ⊗ = Outlier ± 2 SD from the treatment group mean not included in analysis for this study or excluded due to technical error in the Western blot procedure (last well). Data were analyzed by Two-Way ANOVA for main effects (treatment or drug) and interaction (treatment x drug). *P* < 0.05 was considered statistically significant. # = effect of treatment (Normoxic vs. Hypoxic) and * = effect of drug (Saline vs. Vitamin C). All significant results presented are main effects.

There was no significant effect of chronic fetal hypoxia or antenatal Vitamin C on the number of SP-B producing cells in the alveolar epithelium in the lung of young adult lambs (Figures 4A–G). Chronic fetal hypoxia increased gene expression of *ELN*, while antenatal Vitamin C normalised its expression (Figure 4H). There was no significant effect of chronic fetal hypoxia or antenatal Vitamin C on elastin protein expression (Figure 4I) or mRNA expression of *COL1A1* or *MMP-9* (Table 3) in the lung of young adult lambs.

## 4 Discussion

The data in this model show that chronic fetal hypoxia in the last third of pregnancy reduces birth weight, but lambs born from hypoxic pregnancy catch up their growth by young adulthood. Maternal treatment with Vitamin C in hypoxic pregnancy did not rescue birthweight or affect the weight of the female young adult lambs. Further, the data show that chronic fetal hypoxia and antenatal Vitamin C have persisting effects on the molecular regulation of oxidative stress, mitochondrial function, surfactant maturation, inflammation, airway remodelling and hypoxia and glucocorticoid signalling in the lung of female young adult lambs.

FGR and postnatal catch-up growth is a well-established phenotype in pregnancy complicated by chronic fetal hypoxia in various animal models (Giussani and Davidge, 2013; Giussani, 2021). Similar effects of adverse pregnancy on fetal and postnatal growth have been reported in humans (Gluckman et al., 2008). Using the same ovine model, we previously reported that chronic fetal hypoxia in the last third of pregnancy decreases body weight at term in the male fetus, and that maternal treatment with Vitamin C in hypoxic pregnancy rescues this effect (Brain et al., 2019). In this sheep model, past and present evidence supports a differential effect of antenatal Vitamin C treatment in hypoxic pregnancy rescuing fetal growth in male but not female offspring. Studying the effect of maternal asthma during human pregnancy on fetal growth has also highlighted sex-specific differences. Whereas the female fetus adapts to the adverse intrauterine environment reducing its growth rate, the male fetus continues to grow normally (Clifton and Murphy, 2004). Therefore, it is possible that the strategy of the female fetus to budget resources and reduce its growth in hypoxic pregnancy overwhelms any protective effect of antenatal Vitamin C, providing an explanation for persisting low birth weight in female lambs whose mothers received antioxidant treatment in the present study.

The induction of oxidative stress is a common response to chronic hypoxia in fetal life in many organs (Giussani and Davidge, 2013; Giussani, 2021). In the present study, data on the lungs of young adult female lambs show that chronic fetal hypoxia has persisting pro-oxidant effects increasing *NOX-4* expression, and that maternal antenatal treatment with Vitamin C in hypoxic pregnancy neutralizes this effect, likely by maintaining the upregulation of antioxidant pathways into adulthood, such as those stimulated by catalase.

High-altitude pregnancy promotes pulmonary hypertension in offspring (Heath-Freudenthal et al., 2022), and previous studies have reported protective effects of the haemoxygenase-carbon monoxide (HO-CO) pathway in the lungs of newborn llamas and sheep (Herrera et al., 2008; Llanos et al., 2012). This pathway promotes potent pulmonary vasodilator mechanisms mediated by the generation of CO, an endogenous gas synthesized by HO, which acts like nitric oxide to promote vasodilatation in the pulmonary vascular bed. Therefore, a fall in *HMOX-1* expression in the lung of young adult lambs from hypoxic pregnancies in the present study suggest programming of reduced vasoactive mechanisms to protect against pulmonary hypertension. Restoration by antenatal Vitamin C treatment in hypoxic pregnancy suggests protection against this programming effect. However, these ideas require testing in future experiments. Other data in the present study show that exposure to antenatal Vitamin C in hypoxic pregnancy increased key hypoxia signalling factor *HIF-2α* gene expression and decreased *EGLN-2* gene and respective PHD-1 protein expression in the lungs of young adult female lambs born from normoxic or hypoxic pregnancy. This may confer an advantage for the Vitamin C exposed lambs to a hypoxic challenge in postnatal life by enhancing the lung’s response to hypoxia (McGillick et al., 2016b). Interestingly, lungs of lambs exposed to antenatal Vitamin C had reduced protein expression of oxidative phosphorylation (OXPHOS) complexes 1, 2 and 3, despite no change in mitochondrial abundance protein expression, suggesting complex-specific decreases in mitochondrial respiration capacity (Andreyev et al., 2005). A further mechanism may include remodeling of the mitochondrial structure, as Vitamin D supplementation has been shown to alter mitochondrial size and cristae-shaping protein expression levels (Ren et al., 2020; Quigley et al., 2022). In the context of hypoxic insult, changes to mitochondrial structure and intraorganellar connections may aid in the diffusion of the local harmful effects of reactive oxygen species (Moltedo et al., 2019). Since complexes 1 and 2 are the main site for free radical generation in mitochondria, a fall in their activity in different organs subjected to hypoxia has been described as a well-established endogenous antioxidant defence to limit free radical synthesis (Heather et al., 2012; Colleoni et al., 2013; Horscroft et al., 2017). Therefore, this represents a further mechanism via which maternal antenatal treatment with Vitamin C may protect organs of offspring against oxidative stress (Orgeig et al., 2015; McGillick et al., 2016a; McGillick et al., 2016b).

In the present study, the lungs of female young adult lambs exposed to antenatal Vitamin C had increased gene expression of the glucocorticoid activating enzyme *HSD11B-1*. This may represent a compensatory mechanism to overcome the reduced protein expression of 11βHSD-1 in the lung to maintain normal lung tissue cortisol concentration. Exposure to antenatal Vitamin C increased gene expression of inflammatory marker *IL-1B*, but exposure to neither maternal chronic hypoxia or Vitamin C affected *TGFB1* expression. While molecular signalling pathways regulating inflammation are complex and only gene expression of two markers have been investigated in this study, the findings provide insight into effects of antenatal Vitamin C exposure on genes regulating inflammation in the lung in postnatal life. This finding complements the evidence for Vitamin C as a regulator of hypoxia signalling and immune cell function in inflammation and cancer (Ang et al., 2018). Furthermore, changes in pulmonary inflammation status have been shown to reflect altered local glucocorticoid availability (Du et al., 2017; Bansal et al., 2022). Taken together the findings in this study demonstrate effects of antenatal Vitamin C on molecular regulation of glucocorticoid signalling and inflammation, two key pathways supporting normal lung function.

Unlike changes observed in fetal life in experiments using this ovine model of late-onset hypoxic pregnancy (McGillick et al., 2017; McGillick et al., 2022; McGillick et al., 2025, data in the present study show no persisting effect of chronic fetal hypoxia or antenatal Vitamin C on gene expression of surfactant proteins, likely due to all lambs being raised in normoxic conditions from birth. In contrast, the increased expression of surfactant lipid transporter *ABCA3* following exposure to chronic fetal hypoxia persisted into early adulthood (McGillick et al., 2017). However, there was no difference in ABCA3 protein expression, which suggests that this may represent a pool of gene expression to aid in surfactant lipid transport in response to hypoxia.

While there was no significant effect of either chronic fetal hypoxia or antenatal Vitamin C on the number of cells producing SP-B in the alveolar lung tissue of lambs in early adulthood, there was a significant upregulation of airway remodelling marker *ELN* in lambs exposed to chronic hypoxia *in utero* and this effect was normalized in hypoxic pregnancy treated with Vitamin C. These findings support the long-term effects of hypoxic pregnancy on airway remodelling in young adulthood promoted by oxidative stress and successful therapy against it using maternal antenatal treatment with antioxidants. While this study builds upon previous findings of the impact of maternal chronic hypoxia and antenatal Vitamin C exposure in fetal life, the fetal studies investigating outcomes in male fetuses and postnatal studies investigating outcomes in female fetuses mean that direct comparison between studies cannot be made. A further limitation of this study is that lamb lung tissue was immersion fixed and not perfusion fixed, thus we were unable to perform stereological analysis on airway structure and elastin deposition. As a result, we cannot confirm if the upregulation of *ELN* in the lungs of young adult lambs in response to chronic fetal hypoxia is due to thickening of the lung tissue, as in chronic lung disease and/or due to increased alveolarization/vascularization. Further, as this study used tissue collected as part of a primary study investigating cardiovascular physiology in young adult lambs, lung function measurements were not performed. Therefore, we are unable to investigate if molecular changes in pathways triggered by hypoxic pregnancy with and without antenatal Vitamin C treatment translate into functional outcomes. Given the significant effects of exposure to these antenatal insults evident in early adulthood and likely sexual dysmorphic effects, it would be valuable in the future to investigate molecular signalling pathways coupled with functional outcomes in male and female offspring of normoxic and hypoxic pregnancy treated with antioxidants.

## 5 Conclusion

The findings of this study support the hypothesis that chronic fetal hypoxia and antenatal Vitamin C exposure impact factors regulating molecular signalling in the lamb lung in early adulthood and highlight both persistent and differential effects of exposure to chronic fetal hypoxia and antenatal Vitamin C treatment in the lungs of female young adult lambs. This body of work adds to our understanding of the effects of chronic fetal hypoxia and antenatal antioxidant interventions on lung physiology and provides important considerations for the evaluation of potential antioxidant intervention in the fetal, newborn and postnatal periods of life.

## Data Availability

The datasets presented in this study can be found in online repositories. The names of the repository/repositories and accession number(s) can be found in the article/supplementary material.
